# Enhancing maritime transportation security: A data‐driven Bayesian network analysis of terrorist attack risks

**DOI:** 10.1111/risa.15750

**Published:** 2024-07-21

**Authors:** Massoud Mohsendokht, Huanhuan Li, Christos Kontovas, Chia‐Hsun Chang, Zhuohua Qu, Zaili Yang

**Affiliations:** ^1^ Faculty of Engineering and Technology, Liverpool Logistics, Offshore and Marine (LOOM) Research Institute Liverpool John Moores University Liverpool Merseyside UK; ^2^ Liverpool Business School Liverpool John Moores University Liverpool Merseyside UK

**Keywords:** Bayesian network, Global Terrorism Database, maritime terrorism, security risk assessment

## Abstract

Maritime terrorist accidents have a significant low‐frequency‐high‐consequence feature and, thus, require new research to address the associated inherent uncertainty and the scarce literature in the field. This article aims to develop a novel method for maritime security risk analysis. It employs real accident data from maritime terrorist attacks over the past two decades to train a data‐driven Bayesian network (DDBN) model. The findings help pinpoint key contributing factors, scrutinize their interdependencies, ascertain the probability of different terrorist scenarios, and describe their impact on different manifestations of maritime terrorism. The established DDBN model undergoes a thorough verification and validation process employing various techniques, such as sensitivity, metrics, and comparative analyses. Additionally, it is tested against recent real‐world cases to demonstrate its effectiveness in both retrospective and prospective risk propagation, encompassing both diagnostic and predictive capabilities. These findings provide valuable insights for the various stakeholders, including companies and government bodies, fostering comprehension of maritime terrorism and potentially fortifying preventive measures and emergency management.

## 1 INTRODUCTION

Due to the immense significance of global maritime trade, constituting over 80% of worldwide trade in goods, there are notable concerns regarding the potential impact of terrorist attacks. Given the vast expanse and relatively unregulated environment of seas and oceans, maritime transportation has emerged as an appealing target for terrorist groups. However, in comparison to incidents on land, acts of maritime terrorism represent a minimal proportion of overall armed violence and terrorist attacks, as indicated by Asal and Hastings (2015). The Global Terrorism Database (GTD) reports that less than 0.2% of the total attacks have occurred at sea in the last five decades (National Consortium for the Study of Terrorism and Response to Terrorism [START], 2022). Nonetheless, this statistical rarity does not warrant overlooking or underestimating the potential consequences of maritime terrorism. Before further discussing the intricacies of maritime terrorism, it is essential to establish a clear definition of the term. It should be noted that, similar to terrorism, there is no universally accepted definition for maritime terrorism (Zelenkov et al., 2022). The GTD (2021) defined a terrorist attack as “the threatened or actual use of illegal force and violence by a non‐state actor to attain a political, economic, religious, or social goal through fear, coercion, or intimidation.” Nevertheless, given that maritime terrorism falls within the broader category of terrorism, its general definition should align with that of terrorism. However, when considering the specific aspects of the definition, it is important to acknowledge that maritime terrorism possesses distinct features of its own. The term “maritime terrorism” is employed to encompass acts of terrorism that arise from the complexities associated with maritime security.

According to Nincic (2005), maritime terrorism is defined as “any illegal act directed against ships, their passengers, cargo or crew, or against seaports with the intent of directly or indirectly influencing a government [or] group of individuals.” Joyner (1989) has presented a somewhat different definition that extends to encompass threats originating from a vessel associated with terrorist activities. It describes maritime terrorism as “the systematic use or threat to use acts of violence against international shipping and maritime services by an individual or group to induce fear and intimidation in a civilian population in order to achieve political ambitions or objectives.” Concerns regarding methodological approaches to defining “maritime terrorism,” the potential threats posed by maritime terrorism, and strategies to address its challenges are also discussed in Asal and Hastings (2015), Farrell (2007), Nincic (2005), and Knyazeva and Korobeev (2015). Drawing insights from an extensive collection of articles, it can be inferred that maritime terrorism essentially entails a manifestation of political violence employing a strategic approach aimed at destabilizing or disrupting maritime transportation processes to attain specific political objectives.

Within the realm of maritime terrorism, a plethora of maritime targets exist, including but not limited to a variety of ship types, such as oil tankers, cargo ships, fishing boats, LNG carriers, ferries, naval crafts, and civilian vessels. In terms of infrastructures, container seaports and harbors with oil and LNG terminals, refineries, petrochemical installations, underwater pipelines, seabed‐crossing cables, and offshore facilities pose attractive targets for terrorist groups. In this context, it is important to highlight the susceptibility of maritime targets. Actions, such as armed assaults against passenger vessels, detonating oil tankers, abducting ship crew, or hijacking cargo vessels, could prove quite impactful, capturing global media attention and providing terrorists with a significant advantage. Events like the destruction of a refinery or a major port, the sinking of a ship, and the obstruction of maritime chokepoints can hold considerable political value for terrorists and have the potential to inflict substantial economic losses at both national and international levels.

From a different perspective, terrorists require substantial funds to fuel their nefarious objectives. One lucrative avenue for acquiring financial resources involves hijacking cargo ships and kidnapping crew members, subsequently demanding ransom. Additionally, it is crucial to consider the potential for engaging in activities that could result in environmental harm. This includes attacking containers transporting hazardous materials, such as nuclear waste, contaminated materials, or other chemical‐related substances (Rajput, 2022). Such actions not only pose security threats but also have the potential to cause significant environmental damage, as demonstrated by the most heinous incidents, such as Achille Lauro in 1985 and the USS Cole in 2000 (START, 2022). In contrast to maritime incidents driven by factors, such as human errors, equipment failure, or environmental conditions, maritime terrorism poses a distinct challenge. It involves intelligent human actors or actors who take extreme risks and do not follow standard procedures, can bypass security measures, and are notably driven by the desire to attract media attention. Nevertheless, documented incidents indicate that although the tactics employed by terrorists have experienced minimal change, there has been a consistent expansion in the range of targets they choose to attack.

This article, therefore, aims to develop a new maritime security risk analysis method. It will use real data from maritime terrorism incidents over the past two decades to train a data‐driven Bayesian network (DDBN) model. Note that the scope of this study is limited to attacks against ships rather than infrastructure such as seaports or offshore facilities. It seeks to track recent trends in maritime terrorism and identify potential patterns. The article also pinpoints influential factors in maritime terrorism, such as vulnerable regions, high‐risk countries, types of weapons, and attack tactics, and aids to discern the causal relationships among these factors. Moreover, to the best of our knowledge, this article formulates the first comprehensive methodology for conducting a quantitative terrorism risk analysis in the maritime sector. The approach relies on a DDBN and leverages information accumulated over the last two decades from the GTD. This methodology facilitates a comprehensive evaluation of the risks linked to terrorist attacks, providing a robust foundation for in‐depth analysis. Ultimately, this article provides valuable insights for the maritime community on this crucial issue.

The rest of the article is organized as follows. Section 2 offers a comprehensive critique of the current literature on maritime terrorism and the utilization of the technique. In Section 3, the specifics of the chosen methodologies are investigated, encompassing the procedures of data collection, Bayesian Network (BN) structure learning, and model validation. Section 4 presents the analysis results and deliberates on the model's outputs. Lastly, the conclusions are drawn in Section 5.

## 2 LITERATURE REVIEW

The persistent challenge of international terrorism remains a concern, with ongoing reports underscoring its high impact on maritime activities. An extensive examination of the literature indicates that studies on maritime terrorism predominantly focus on theoretical frameworks and definitions (Chalk, 2008; Ganor, 2002; Schwenkenbecher, 2012), governmental and legal perspectives (Asal & Hastings, 2015; Hong, 2012; Rajput, 2022; Schneider, 2013; Shah, 2013; Tan, 2012), general and private security challenges (Greenberg et al., 2006; Knyazeva & Korobeev, 2015; Nincic, 2005; Richardson, 2004; Tertia & Perwita, 2018; Tilly, 2004), enhancements to operational systems (Knyazeva & Korobeev, 2015; Kuhn et al., 2023), and other qualitative research (Hacaga, 2020; Schneider, 2020; Zelenkov et al., 2022). The majority of past investigations into maritime terrorism have primarily employed a qualitative perspective, with only a limited number delving into the identification of security risk factors that impact maritime terrorism. In research carried out by Nelson (2012), the study identified factors that distinguish maritime terrorism from piracy and also examined whether the individuals responsible for these activities are collaborating with each other. A quantitative assessment of the effects of terrorism and piracy was conducted on maritime‐related economic activities in the Niger Delta region of Nigeria (Eberechukwu Onwuegbuchunam et al., 2021). Using the linear regression analysis model, the authors identified a noteworthy inverse relationship between the chosen maritime‐related economic activities and incidents of maritime terrorism and piracy. Statistically examining the period from 2010 to 2017, Schneider (2020) conducted an analysis of terrorist attacks on maritime targets. This research investigates the current patterns in global maritime terrorism, the attributes of the perpetrators and their attacks, the geographical regions of the incidents, and the types of weapons and methods used.

Although quantitative studies in the maritime industry related to terrorism are scarce, other domains have employed a diverse range of quantitative analysis models to scrutinize terrorism (Dillon et al., 2009; Li & Yang, 2023; Liang et al., 2024; Monroe et al., 2018; Regens et al., 2015; Rezazadeh et al., 2019). Ezell et al. (2010) investigated various methodologies for analyzing security risks of terrorism, with a focus on probability and decision‐making frameworks. The methods explored included fault/attack/success tree analysis, event tree analysis, and game theory. Nevertheless, these approaches exhibit weaknesses in modeling terrorist risk. They struggle to handle uncertainties associated with influential factors and cannot clearly pinpoint the causal interrelationship among these factors (Zhu et al., 2020). Moreover, they face challenges in addressing questions regarding the specific impact and level of influence that different factors have on the varied attack risks posed by individual perpetrators. Given the drawbacks highlighted earlier, BN emerges as a promising method for overcoming these limitations. BN has the capability to incorporate information from multiple sources, assess the likelihood of events, forecast the outcomes of diverse scenarios, account for both objective and subjective data, address variables with multiple states, handle multiple outputs, and identify causal relationships among contributing factors leading to the top event. BN also possesses a robust theoretical foundation and is well suited for handling incomplete data. Additionally, it exhibits the ability to incorporate new data, updating the probabilities of events accordingly (Kabir & Papadopoulos, 2019; Wei, 2022). When employing BN for risk assessment in safety or security, the initial step involves constructing the BN model, which includes multiple interdependent risk factors. Various methods can be utilized for this purpose, such as literature review, expert judgment, data learning, or a combination of these approaches. In the realm of maritime risk assessment, Bouejla et al. (2014) and Pristrom et al. (2016) employed expert judgment along with data sourced from the International Maritime Organization (IMO) to establish a BN structure for assessing the risk of piracy attacks on ships. Similarly, Jiang and Lu (2020) utilized a combination of statistical data and expert knowledge for BN structure learning, applying this approach to analyze maritime piracy in Southeast Asia.

Although expert knowledge remains crucial for BN development, particularly in situations with incomplete or unavailable data, it is acknowledged that this approach introduces subjectivity and potential uncertainty. To mitigate such subjective elements, there is a growing trend in the academic community toward DDBN approaches. This methodology becomes particularly popular when there is a wealth of data available, allowing for the construction of BN models based on learning from the data itself. The utilization of a data‐driven approach is evident in various maritime risk assessment studies. Some researchers employed a DDBN model to simulate global maritime risk analysis (Li et al., 2023), investigate collision risk analysis (Li, Çelik et al., 2024), explore the dynamic accident evolution (Li, Zhou et al., 2024), and conduct maritime severity analysis (Zhou et al., 2024). Fan et al. (2022) developed a DDBN model to explore the joint impact of risk factors on different types of maritime accidents within restricted waters. In another instance, Liang et al. (2022) conducted an analysis of influential risk factors affecting theft‐related accidents in freight supply chains. They constructed a BN using a data‐driven approach to predict the likelihood of the occurrence of such events.

In summary, the main research gaps identified from the literature review are summarized as follows:

(1) Predominance of qualitative studies: Most previous research on terrorism broadly, and maritime terrorism specifically, has predominantly focused on qualitative analysis. These include theoretical frameworks, governmental and legal viewpoints, and overall security challenges. There is a noticeable lack of quantitative analysis of the specific security risk factors affecting maritime terrorism.
(2) Limitations with current quantitative approaches: Conventional approaches for assessing terrorism‐related security risks, including fault/attack/success tree analysis, event tree analysis, and game theory, have notable limitations. These techniques have difficulty managing uncertainties and are not adept at uncovering causal interrelationships among the various influential factors.
(3) Incompleteness of maritime terrorism studies: Many current studies in this field fail to encompass the spatial–temporal aspects of recorded attacks. This results in an incomplete understanding of the actual events.
(4) Shortage of data‐driven approaches: There is a growing trend toward DDBN approaches in risk science, which are particularly useful when there is a wealth of data available. These approaches can construct BN models based on data learning, reducing the subjectivity associated with expert judgment. Although DDBN models hold promise, their use in maritime security risk assessment is still limited, presenting opportunities for further investigation.

Building upon prior research and the research gaps identified, this article endeavors to pioneer the application of data‐driven learning in constructing a Tree‐Augmented Naïve Bayes (TAN)‐based model for the analysis of maritime terrorism. To achieve this objective, the GTD database is used to extract information on terrorist incidents involving ships over a 22‐year period from 2000 to 2021. The outcomes of this study aim to provide a substantial contribution to the comprehension of maritime terrorism. Additionally, the research seeks to enhance understanding of the risk characteristics associated with terrorist attacks in this domain, filling a gap that, to the best of the authors’ knowledge, exists in the current literature.

## 3 METHODOLOGY

**FIGURE 1:**
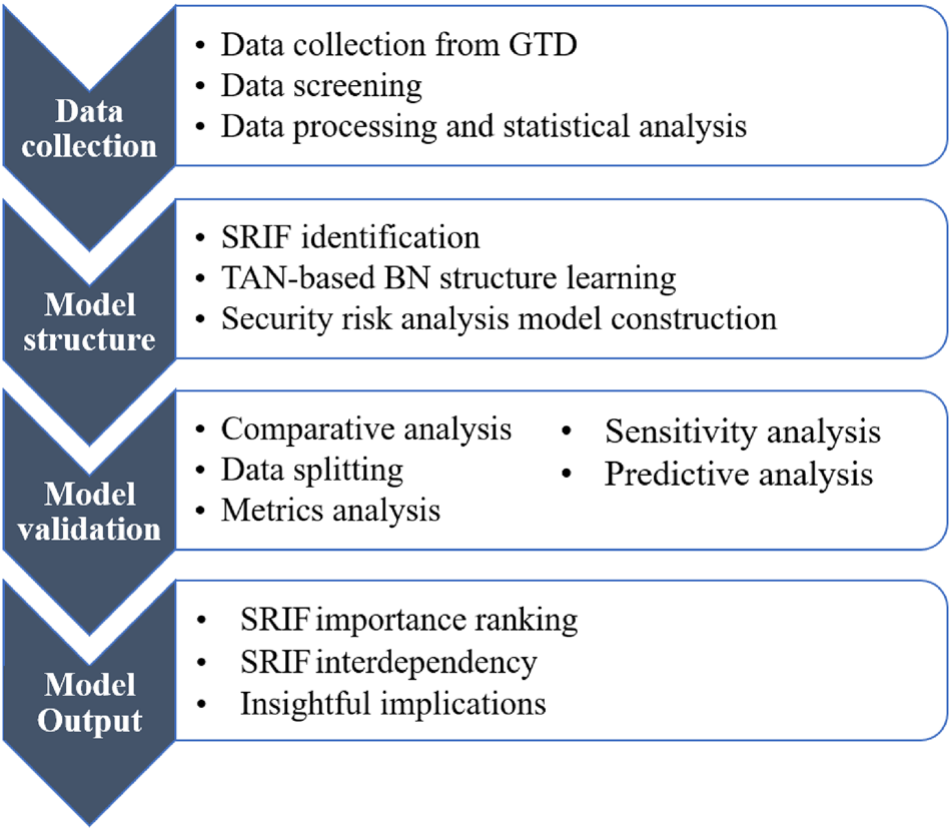
The proposed methodology framework.

In this research, a DDBN technique is employed to uncover the Security Risk–Influencing Factors (SRIFs) affecting terrorist incidents in maritime transportation. The primary goal is to assess the significance of these factors and gain a more profound insight into the workings of maritime terrorism. This methodology comprises four primary stages, namely, data acquisition and processing, model construction and development, model validation, and model output. Figure 1 depicts the comprehensive framework of the proposed methodology.

### 3.1 Data collection, exploration, and processing

This study utilizes the GTD as its main source for documenting terrorist incidents. Initially, alternative terrorist databases were considered, including the National Memorial Institute for the Prevention of Terrorism (MIPT) Terrorism Knowledge Base, the Worldwide Incidents Tracking System (WITS), the Research and Development (RAND) Database of Worldwide Terrorism Incidents, and the International Terrorism: Attributes of Terrorist Events (ITERATE). Nevertheless, the GTD is notable for being the most extensive repository of terrorism incidents because of the following reasons: (a) The prior databases have been discontinued for a considerable duration of time; (b) they do not possess the metadata accumulated by the GTD compilers; (c) different databases offer varying definitions of terrorism. Contrary to other databases, GTD is an extensive compilation of reported terrorist activities worldwide, encompassing over 200,000 incidents since 1970. The data are sourced from the US START, an esteemed department of Homeland Security of Excellence located at the University of Maryland in the United States (*START*, 2022). The first step involved extracting the pertinent data on maritime terrorism from the GTD for the period from 2000 to 2021. It should be noted that the GTD has not recently updated its database over the past 2 years, but it is still the most comprehensive database available. Out of the 204 identified occurrences, 160 cases are specifically linked to acts of terrorism targeting ships and other marine vehicles. The remaining incidents consist of terrorist assaults targeting seaports and other fixed infrastructure, which have been excluded from the analysis.

**FIGURE 2:**
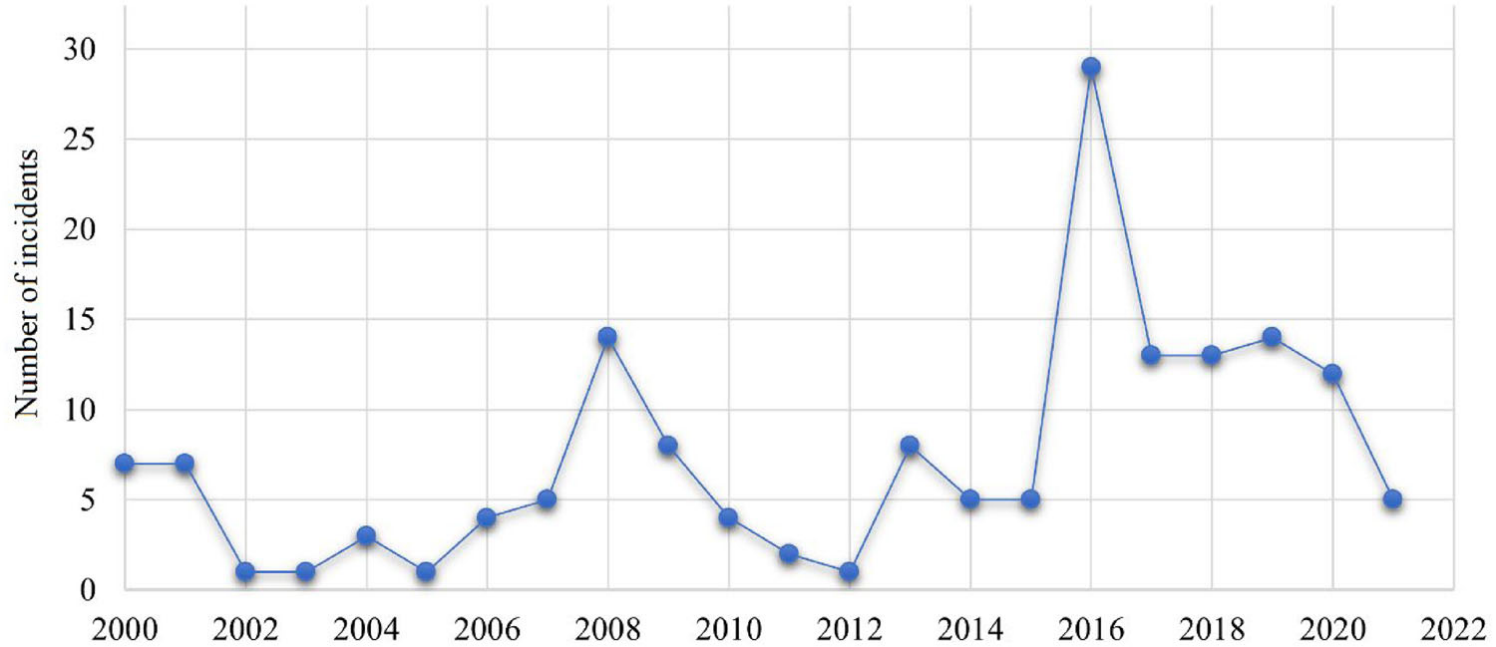
The distribution of terrorist attacks for maritime vessels over 22 years.

**FIGURE 3:**
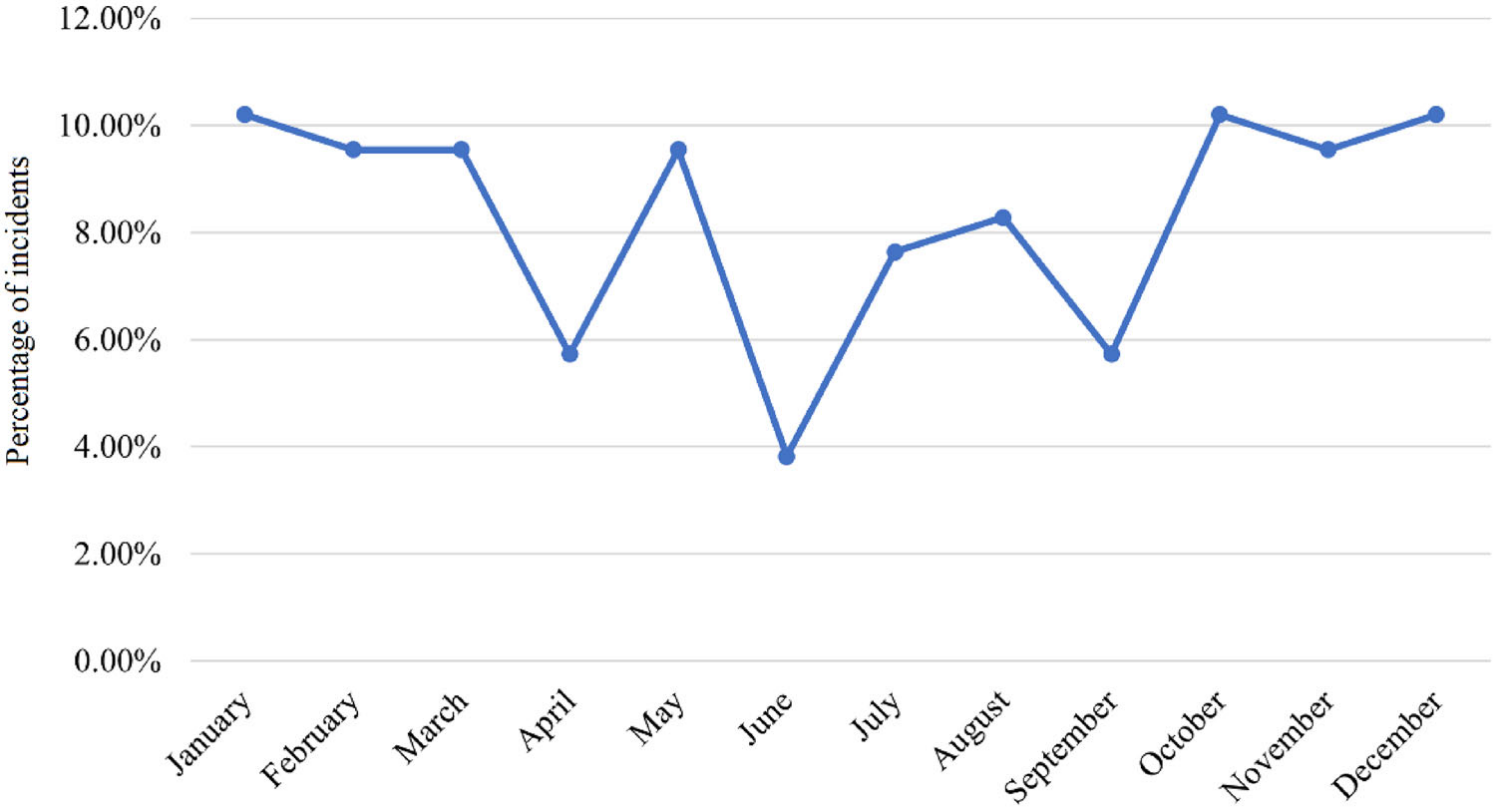
The percentage of incidents over months of year.

**FIGURE 4:**
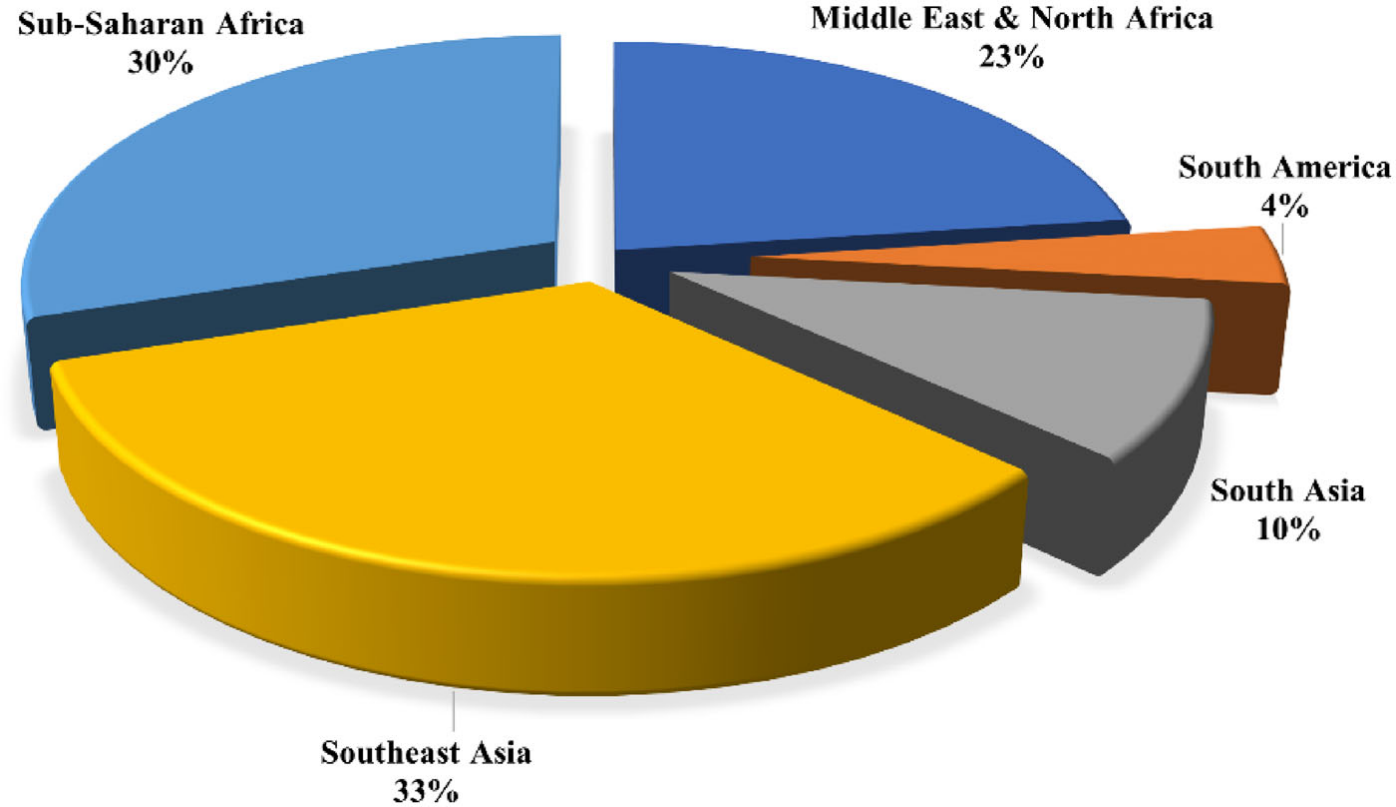
The distribution of incidents over critical regions of the world.

**FIGURE 5:**
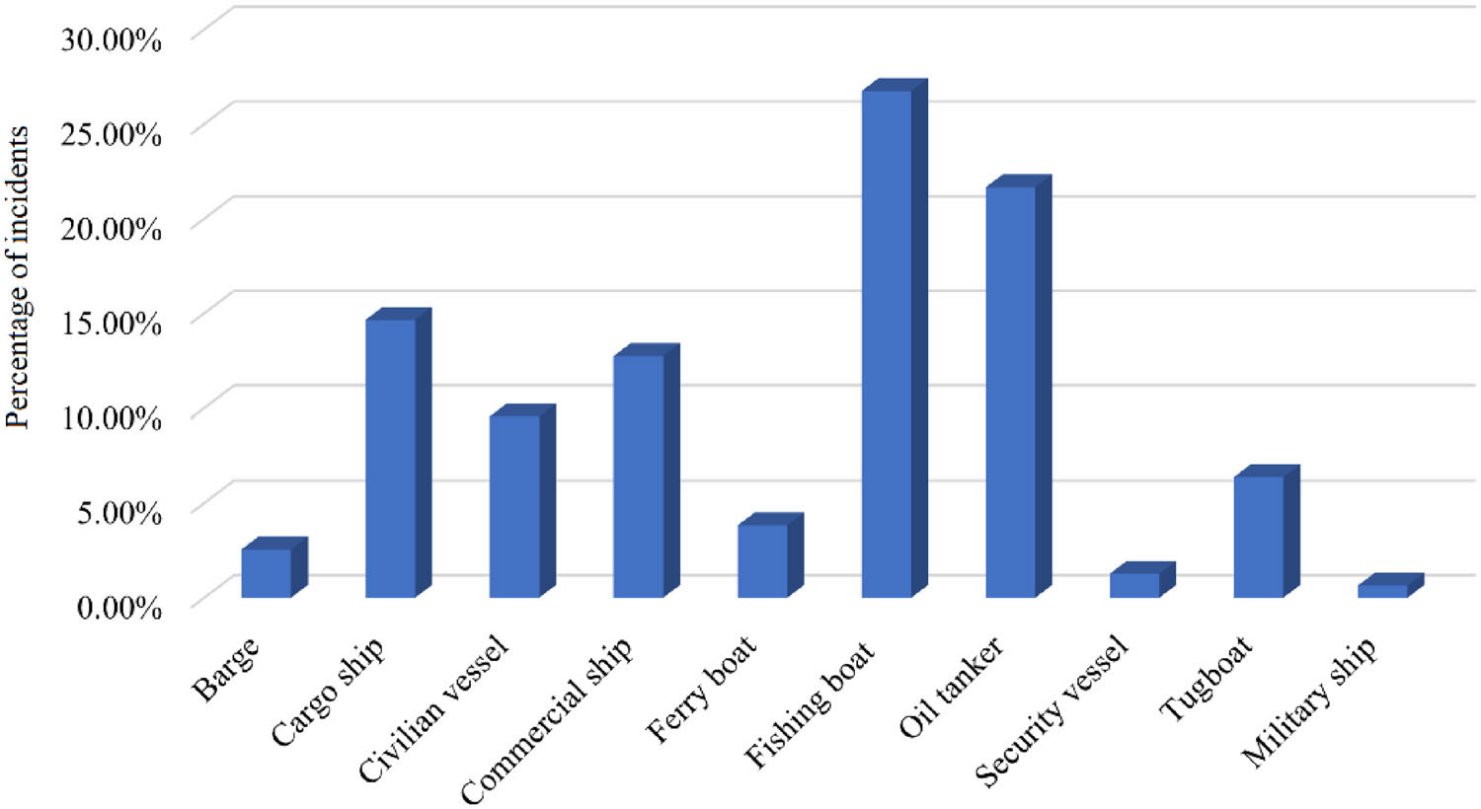
The percentage of incidents for different types of vessels.

The GTD encompasses a multitude of characteristics pertaining to terrorist occurrences, such as the nature of terrorism, the regions most affected by terrorism, the nations where the incidents took place, and the dates of the incidents. There are numerous aspects related to terrorism that prompt us to analyze the spatial–temporal distribution of maritime terrorism. As depicted in Figure 2, the frequency of terrorist attacks on ships does not exhibit a discernible pattern. Nevertheless, there has been a general increase in the number of incidents over the last 10 years, reaching a peak in 2016. Figure 3 illustrates the distribution of terrorism occurrences by month, revealing that the first and last 3 months of the year witness the highest incidents. Five main global locations are focal points for maritime terrorist activity, as depicted in Figure 4. Southeast Asia and Sub‐Saharan Africa are particularly identified as high‐risk regions. An additional noteworthy aspect is the variety of maritime vessels susceptible to terrorism. Figure 5 shows that fishing boats and tankers are the primary targets for terrorist activities. Following closely are cargo and commercial ships. It is important to note that various vessels may be vulnerable to different types of terrorist attacks, a topic that will be explored in the subsequent sections.

### 3.2 Analysis of SRIF on maritime terrorist attacks

In this article, the security‐affecting factors are referred to as SRIFs. These factors are determined by analyzing data from the GTD, integrating with the information obtained from the literature review to categorize and compile the relevant indicators. To accurately evaluate the risk level of terrorist attacks and identify the factors involved, it is necessary to carefully investigate elements, such as the date, location, methods of attack, types of weapons employed, casualties, degree of damage, and the overall impact of the incidents. Following the outlined procedures, a total of 12 distinct SRIFs were identified, including factors such as the type of attack, ship category, weapon employed, region, country, flag state, year, perpetrator, success of the attack, property damage, ransom, and casualties.

Table 1 provides details on various terrorist attack types, whereas Table 2 delineates the remaining SRIFs, offering descriptions and the associated states linked to each.

**TABLE 1:** Types of terrorist attacks against maritime transportation (GTD, 2021).

Attack type	Description
Assailment	A deliberate attack with the intention of leading to physical harm or causing death to others using weapons, incendiary devices, or sharp implements (such as knives). It also includes attacks involving particular categories of explosive devices, such as grenades, projectiles, and unidentified thrown explosive devices, as well as weapons, incendiaries, or cutting tools
Explosion	This refers to attacks utilizing unstable materials that rapidly release energy, creating a pressure wave causing physical harm to the environment. This release can result from chemical reactions, high pressure, or other processes, leading to a sudden and forceful release of energy, often causing damage to its surroundings. It covers both high and low explosives, including dirty bombs, but excludes nuclear devices
Hijacking	Hijacking refers to a deliberate action with the objective of taking control of a marine vessel in order to divert its course or achieve political objectives. Although ransom may not be the only purpose, it could be present in conjunction with other stated objectives. Hijacking pertains to the act of forcibly taking control of a ship, as opposed to hostage taking, which mostly involves the abduction of individuals rather than the vehicle itself
Hostage taking (kidnapping)	Kidnappings are defined as the deliberate act of taking hostages with the aim of achieving political objectives or causing disruption to regular activities. Kidnappings differ from barricade incidents in that they entail the act of forcibly moving and confining individuals against their will. The main objective is to gain political goals by either making concessions or disrupting regular activity for a specific reason. Occasionally, a ransom may also be demanded

**TABLE 2:** Description of Security Risk–Influencing Factors (SRIFs) and their states.

Node number	SRIFs	States	Description
1	Ship type	Cargo ship, civilian vessel, commercial ship, fishing boat, tanker, tugboat, other	“Other” encompasses barges, military vessels, security ships, and ferries. Tankers refer to oil, gas, and chemical carriers
2	Weapon type	Bomb, combinatory, explosive‐laden boats, firearms, projectile, unknown	“Bomb” encompasses naval mines, suicide bombers, time fuses, dynamite/TNT, sticky bombs, pipe bombs, and other unidentified explosive types “Combinatory” refers to employing a combination of weapons, for instance, utilizing projectiles to damage ships, followed by employing firearms to attack them “Explosive‐laden boats,” often referred to as suicide boats or explosive boats, are watercraft that have been loaded with explosives with the intention of being used as a weapon. These boats loaded with explosives can be operated either by individuals onboard (suicide mission) or controlled remotely from a distance “Firearms” includes all portable weapons, such as automatic or semi‐automatic rifles, handguns, rifles/shotguns, and any other unclassified gun types “Projectile” refers to various items like rockets, mortars, RPGs, and missiles
3	Region	Middle East and North Africa, South America, South Asia, Southeast Asia, Sub‐Saharan Africa	These are the sole regions globally where terrorist attacks against maritime transportation have been documented
4	Country	Cameroon, Colombia, Congo, India, Iraq, Libya, Malaysia, Mozambique, Myanmar, Nigeria, Philippines, Saudi Arabia, Somalia, Sri Lanka, Yemen, other	Countries where the terrorist attacks occurred fewer than three times are grouped into the “other” category
5	Flag state	China, Colombia, Congo, Egypt, Greece, India, Indonesia, Iran, Iraq, Italy, Japan, Liberia, Malaysia, Mozambique, Multinational, Myanmar, Netherlands, Nigeria, Pakistan, Philippines, Romania, Russia, Saudi Arabia, Sierra Leone, South Korea, Spain, Sri Lanka, Thailand, Turkey, the United States, Vietnam, Yemen, other	The ship's flag state is the nation where the ship is registered, which may not align with the location of the incident “Other” denotes flag states that have experienced fewer than two attacks
6	Year	2000–2021	–
7	Perpetrator	AA, ALQ, ALS, AMC, ASG, BIFM, FARC, HE, LTTE, ME, MEND, MMCM, NDV, NPA, other, unknown	Terrorist groups are denoted by acronyms, with their complete names accessible on the GTD website. This article features the perpetrators recorded at least twice, whereas the remaining are grouped as “other.” Instances exist where no responsible perpetrator was identified, labeled “unknown”
8	Successful attack	Yes, no	A successful attack is based on immediate impact, not broader goals; a bomb exploding, even without major consequences, counts as success
9	Property damage	Yes, no	The presence of property damage is indicated by a “yes.” If “property damage” is affirmative, one of the following three categories describes the extent of damage: 1 = Catastrophic (probably ≥$1 billion) 2 = Major (probably ≥$1 million but <$1 billion) 3 = Minor (probably <$1 million) For simplicity, we only take into account whether property damage exists or not
10	Ransom	Yes, no	In cases of hijacking or hostage‐taking incidents, ransom denotes the monetary demand required for the release of kidnapped individuals or the return of hijacked vessels
11	Casualty	Yes, no	Casualties refer to the total count of confirmed fatalities and injuries resulting from the incident, encompassing both victims and attackers who perished directly due to the event

### 3.3 BN structure learning

In building the BN model, there are mainly two approaches: One involves data‐free modeling, relying on expert judgment, and introducing uncertainty and bias, whereas the other employs data‐driven methods. The latter utilizes empirical data to steer the search‐based learning process, yielding objective results. In this article, the focus of BN structure learning is to unveil a directed acyclic graph structure that effectively represents the relationships among SRIFs, which are variables derived from the collected data. These relationships encompass dependencies, interdependencies, or even independence among influential factors.

Various DDBN approaches exist in the literature, including NPC algorithm, K2 algorithm, Naive BN (NBN), augmented BN, Bayesian search, greedy thick thinning, and TAN, each with its own strengths and weaknesses, contingent upon factors, such as the volume of data, data sources, number of nodes, and model validation (Meng et al., 2022). This article has opted for using the TAN approach, an improved version of NBN, to investigate the BN structure learning. In a standard naive Bayes classifier, it is assumed that all features are conditionally independent when given the class variable, a simplification that leads to its characterization as “naive.” The TAN algorithm departs from the assumption of feature independence by introducing dependencies among features, yet it adheres to a tree structure for these relationships. TAN holds an edge over the standard naive Bayes by effectively capturing particular interconnections among features (Friedman et al., 1997). This capability enhances classification accuracy, particularly in scenarios where naive Bayes’ independence assumption proves overly limiting. It therefore witnesses the rising profile of using TAN‐trained BN in maritime‐related risk/safety analysis (Li et al., 2023). The essential steps in TAN modeling can be succinctly outlined as follows (Fan et al., 2020): (a) selecting a target node, typically the class variable, as the initial point for tree construction; (b) creating a maximum weight spanning tree to form connections among the remaining nodes. The edge weights are determined by measuring mutual information among features; (c) defining conditional dependencies: identifying the parent of each feature within the tree. Each feature in the spanning tree structure is conditionally dependent on its parent node and the class variable, given the root node; (d) learning parameters: after establishing the structure, calculate conditional probability distributions for each node based on available data.

Overall, the process of constructing the BN tree structure entails identifying the most informative dependencies while ensuring that it remains acyclic, and the mutual information measure assists in assessing the strength of linkages among variables. TAN achieves a middle ground by combining the simplicity of naive Bayes with the complexity of fully linked BNs.

### 3.4 Model validation

Model validation is the evaluation of the effectiveness and reliability of the constructed BN model. The objective is to guarantee that the model precisely represents the fundamental connections in the data and may provide trustworthy predictions or inferences. In this article, the constructed model undergoes validation using a range of methods, which encompass (a) comparative analysis, (b) data splitting, (c) metrics analysis, (d) sensitivity analysis, and (e) real‐world scenario analysis.

#### 3.4.1 Comparative analysis

A comparative analysis involves assessing the performance and characteristics of a developed BN model by comparing it to other existing models or benchmarks. Various approaches can be employed for this purpose. For example, the BN model is juxtaposed with other models known for their robust performance in analogous tasks. This process facilitates an evaluation of the relative strengths and weaknesses of the BN model compared to other well‐established methodologies. Additionally, the BN model may be contrasted with conventional approaches or algorithms commonly utilized in the industry, providing valuable insights into its performance against widely accepted standards.

In this article, given the limited existing research on maritime terrorism and the scarcity of BN‐based models in this domain, the findings are compared with the initially collected statistical data. In this regard, the predicted probabilities for the states of the target node are contrasted with their associated statistical ones. Greater consistency in the results serves as an indicator of the reliability of the established model (Fan et al., 2022).

#### 3.4.2 Data splitting

The data splitting process in DDBN aids in creating a more reliable, generalizable, and well‐validated predictive model by enabling effective training, testing, and hyperparameter tuning. It replicates the model's performance on data that it has not been exposed to during the training phase. This aids in the detection of potential problems such as overfitting, which occurs when the model exhibits good performance on the training data but fails to generalize to new data. The overall procedure entails splitting the given dataset into two or more distinct subsets: a training set and a validation (or testing) set. The bulk of the dataset, typically 90% of it, is assigned to the training set, where the model is developed and trained. This allocation enables the model to understand the core patterns and relationships within the data. On the other hand, a minor portion of the dataset, usually the remaining 10%, is designated for the validation set. The performance of the model is subsequently assessed on this set to determine its ability to generalize to new, not‐observed data. However, the process of selecting testing data is crucial. Random selection alone may not yield optimal performance. Techniques like *k*‐fold cross‐validation address this by splitting the dataset into *k* subsets (Refaeilzadeh et al., 2009). The model is trained *k* times, each time using a different subset as the testing set and the remaining data as the training set. This approach provides a more reliable estimate of the model's performance and reduces the risk of overfitting. Conversely, underfitting occurs when the model is too simple to capture the underlying patterns in the data. Validating the model on different subsets helps identify and mitigate underfitting issues. Through this data splitting process, the model learns a wider variety of patterns, making it more robust and less sensitive to fluctuations in the data. The higher accuracy rate of the prediction denotes the validity of the model.

#### 3.4.3 Metrics analysis

**FIGURE 6:**
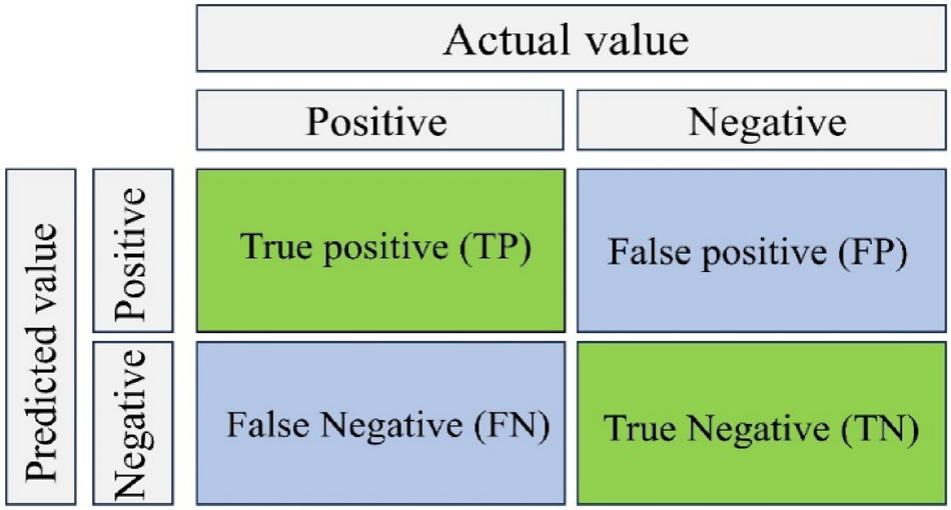
The basic illustration of a confusion matrix.

Metrics analysis is an essential approach for validating BNs as it offers a quantitative evaluation of the model's performance. This approach entails employing diverse measures to assess the degree of alignment between the BN model and the actual data or desired outcomes. In this context, specific metrics have been chosen to assess the performance of the model. The confusion matrix serves as a tabular representation, dividing predictions into categories, such as true positives, true negatives, false positives, and false negatives. Figure 6 illustrates the basic idea of a confusion matrix.

This matrix forms the foundation for calculating several crucial metrics (Simsekler & Qazi, 2022). The overall accuracy metric quantifies the proportion of correctly predicted instances relative to the total instances, offering a comprehensive measure of the model's correctness. Precision, another selected metric, gauges the accuracy of positive predictions by indicating the proportion of true positives among all instances predicted as positive. Furthermore, the recall metric assesses the model's capability to correctly identify all relevant instances, representing the proportion of true positives among all actual positives. Although “precision” evaluates the model's accuracy, “recall” assesses the consistency of the model. The “F1 score,” a metric that strikes a balance between “precision” and “recall,” is computed as the harmonic mean of the two. The harmonic mean is used to assess the overall average distribution. This metric is especially valuable when handling imbalanced class distributions. “Specificity” measures the proportion of correctly predicted negative samples out of all actual negative samples. A higher “specificity” value indicates a better result. Finally, the “False Positive Rsate (FPR),” which is inversely related to “specificity,” a lower FPR value, indicates better performance. The calculation formulas of the above six indicators are shown in the following equations:

(1) $$\mathrm{Overall}\;\mathrm{accurracy}=\frac{T_{P}+T_{N}}{T_{P}+F_{P}+T_{N}+F_{N}},$$

(2) $$\mathrm{precision}=\frac{T_{P}}{T_{P}+F_{P}},$$

(3) $$\mathrm{recall}=\frac{T_{P}}{T_{P}+F_{N}},$$

(4) $$F-\mathrm{measure}=\frac{2\times \mathrm{precision}\times \mathrm{recall}}{\mathrm{precision}+\mathrm{recall}},$$

(5) $$\mathrm{Specificity}=TNR=\frac{T_{N}}{F_{P}+T_{N}},$$

(6) $$FPR=1-TPR=\frac{F_{P}}{F_{P}+T_{N}},$$

where *T_P_* represents the positive samples correctly predicted as positive, and *T_N_* denotes the negative samples correctly predicted as negative. *F_P_* refers to the negative samples incorrectly predicted as positive, whereas *F_N_* represents the positive samples incorrectly predicted as negative. *TNR*, also known as specificity, indicates the true negative rate. Together, these metrics provide a comprehensive evaluation of the model's performance, considering aspects, such as accuracy, precision, recall, and the balance between precision and recall (Hu et al., 2016). This selection enables a thorough assessment of the model's effectiveness in making accurate predictions across different categories and addressing potential challenges posed by imbalanced class distributions.

#### 3.4.4 Sensitivity analysis

In the context of BN analysis, sensitivity analysis refers to the examination of how changes or uncertainties in the probabilities or values assigned to the variables in the network impact the output or results of the model. It is a technique used to assess the robustness and reliability of the BN in the face of variations in the input data. Moreover, it aids in pinpointing the SRIFs that wield the most influence on the target node, enabling the strategic selection of cost‐effective measures to alleviate potential consequences. Various approaches exist for executing sensitivity analysis in BN, such as mutual information, joint probability, True Risk Influence (TRI), and minor variation testing.

“Mutual information,” originating from entropy theory, serves as a metric for evaluating the relationship between two random variables within the BN, quantifying the extent of dependence or shared information between them. Mathematically, the mutual information can be expressed as follows (Li et al., 2023):

(7) $$I\left(A,X_{i}\right)=-\sum_{A,i}P\left(A,\;X_{ij}\right)\mathrm{lo}g_{b}\frac{P\left(A,\;X_{ij}\right)}{P\left(A)P(X_{ij}\right)}.$$

In this context, *A* denotes different attack types on the target node, *X_i_* stands for a random SRIF, *X_ij_* signifies the *j*th state of the *i*th SRIF, *P*(*X_ij_*) represents the probability of the *j*th state of the *i*th SRIF, and *P*(*A*, *X_ij_*) denotes the joint probability of *A* and *X*. Calculating the mutual information provides insights into the relative significance of relevant SRIFs concerning the target node. Typically, a higher mutual information value indicates a stronger association among the variables, offering a gauge of their interdependence.

As commonly practiced in traditional sensitivity analysis, different values are assigned to the states of the nodes under investigation while maintaining the states of other nodes unchanged. This method is generally suitable for nodes that possess only two states. However, when dealing with nodes in a BN model that have multiple states, it becomes challenging to discern how altering one state affects the other states. To address this challenge, a novel method known as TRI, introduced by Alyami et al. (2019) and rooted in joint probability, is employed. The joint probability refers to the probability of a specific combination of states occurring across multiple random variables in the network. The joint probability distribution provides a comprehensive description of the simultaneous occurrences of different states for all the variables in the network. TRI operates in the following manner: Initially, the High‐Risk Influence (HRI) is calculated by raising the probability of the state of an investigated node with the most substantial impact on the target node, to 100%. Following this, the Low‐Risk Influence (LRI) is computed similarly, but the selected state is the one that contributes to the lowest risk stake of the target node. Ultimately, the TRI value for the examined SRIF is determined as the average of HRI and LRI. This procedure can be systematically employed for all SRIFs. A higher TRI value indicates a more pronounced impact of the respective SRIF on the target node. In simpler terms, the target node exhibits greater sensitivity to the SRIF associated with the higher TRI value.

Beyond the sensitivity analysis methods discussed earlier, an additional approach is available for consideration. This method relies on two specified principles (Zhang et al., 2013):

*Principle 1*: A marginal increase or decrease in the prior probabilities of each tested node should result in a proportional increase or decrease in the posterior probability of the target node.

*Principle 2*: The cumulative impact of incorporating probability variations from the evidence should be at least as significant as the impact from a subset of the evidence.

In adherence to these principles, slight modifications to variables are implemented. Following the mutual information results, SRIFs are prioritized, and the updates commence from the less influential nodes, progressively reaching the target node. This process is iteratively carried out for the remaining nodes while retaining the previous outcomes.

#### 3.4.5 Real‐world scenario analysis

To address this matter, several steps are taken. First, a real case scenario is collected from outside of the GTD database to accurately represent the variables within the BN. These data should cover a diverse range of scenarios and conditions. Next, the BN model is applied to make predictions or determinations based on real‐world empirical data. This involves estimating probabilities, predicting outcomes, or making determinations based on the model's structure and parameters. Subsequently, the accuracy of predictions is assessed by comparing them to actual outcomes. The evaluation involves scrutinizing the extent to which the model's predictions align with the observed results in real‐world data. This comparison entails assessing both projected probabilities and observed outcomes, ensuring a comprehensive evaluation of the model's performance.

## 4 RESULTS AND DISCUSSION

### 4.1 TAN modeling

**FIGURE 7:**
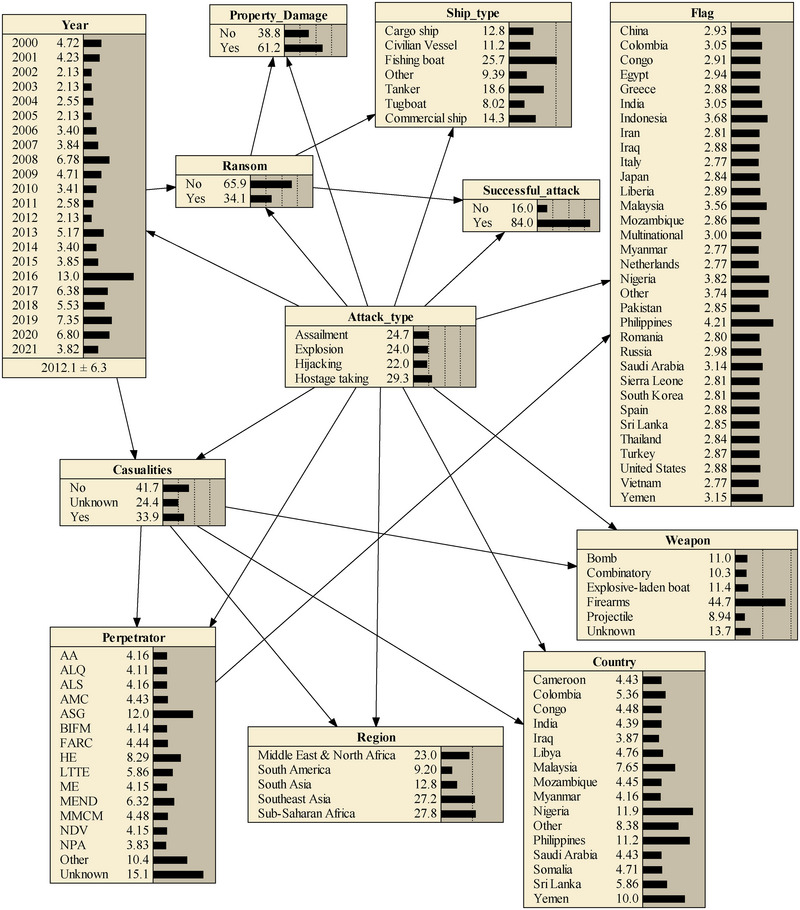
The Tree‐Augmented Naïve Bayes (TAN)‐based Bayesian network (BN) model of terrorist attacks against maritime transportation.

The TAN model is created utilizing data gathered from the GTD database, wherein pertinent details are extracted to identify the SRIF as outlined in Table 2. The model designates the attack type as the target node, and the Netica software (Netica (Version 607)., 2019) is employed to facilitate the construction process. Netica allows in‐depth analytical and statistical assessment of networks with its generous bin capacity. It places no restrictions on the number of nodes and supports dynamically changing values. The graphical interface provides numerical outputs, allowing users to visualize statistics, and offers the flexibility of selecting value ranges as evidence (George & Renjith, 2021). In Figure 7, the depicted process involves the construction of the TAN network, followed by a crucial step in the parameter learning phase. Specifically, this step focuses on refining the parameters associated with the conditional probability tables for different nodes within the network. The refinement process is achieved by incorporating relevant prior data through the Bayesian estimation method. This method adds a layer of sophistication to the learning process, allowing the model to adapt and improve its understanding of the causal relationships and probabilities associated with each node. Essentially, after the TAN network is established, the model undergoes a data‐driven enhancement, fine‐tuning its predictive capabilities through the incorporation of historical data.

As previously noted, the “attack type” is selected as the target node and is therefore directly connected to all other nodes. The rationale behind the constructed connections among different nodes can be interpreted to some extent through expert judgment. Regarding the connection between the target node and success rate, the type of attack can significantly impact the likelihood of success. For instance, certain attack types may have higher success probabilities due to their nature (e.g., explosions might be more successful than hijackings in certain situations). Additionally, the type of attack directly affects the number of casualties, with assailment generally resulting in more casualties compared to hijackings.

In terms of connections between the other nodes, the following examples illustrate the rationality of the constructed model. “Successful attacks,” “property damage,” and “ransom” are all interconnected. If an attack succeeds, the probability of significant property damage and subsequent ransom demands increases, particularly in cases of explosions and hostage‐taking situations. “Casualties” are linked to “weapon,” “region,” “country,” “perpetrator,” and “year.” This multifaceted connection indicates that the lethality and destructiveness of an attack depend on various factors, including its success and nature. The type of weapons used by various perpetrators in different countries and regions worldwide significantly affects the number of deaths and injuries among seafarers or people on ships. For instance, the most dangerous scenarios with high casualties typically involve armed assaults rather than explosions, particularly in the Middle East and Northern Africa. The nationality of a ship (flag) is connected to the perpetrator, suggesting that certain flags might be more susceptible to attacks from specific terrorist groups due to political reasons. By following the connections in the model, it can be seen that it reflects real‐world relationships between various factors involved in terrorist attacks. These connections are crucial for building a comprehensive security risk assessment framework. Each arrow represents a logical dependency that enhances our understanding of the factors contributing to the likelihood and impact of terrorist activities.

### 4.2 Model validation

In accordance with Section 3.4, a variety of approaches are employed to assess the accuracy of the constructed model. These can help determine whether the security risk prediction outcomes accurately correspond to the actual occurrences of maritime terrorism.

#### 4.2.1 Comparative analysis

The TAN model's results, as detailed in Table 3, demonstrate a noteworthy level of consistency and reliability in comparison to historical data. The robustness of the model's predictions is particularly evident, with only minor variations observed. These minor variations between the historical data and TAN results can be explained by two reasons. First, during the data‐splitting procedure, 10% of the data was reserved for alternative validation methods, and the model was constructed using the remaining 90%. Second, the TAN model extends the naive Bayes classifier by incorporating limited dependencies among features, represented as a maximum spanning tree. This approach maintains a balance between simplicity and effectiveness, enhancing computational efficiency and ease of interpretation. Although this can lead to minor discrepancies, such as a 0.2% error between the model's probabilities and actual data, such errors are typically acceptable, as evident by Fan et al. (2020) and Yang et al. (2018). The result reveals that hostage taking is the predominant form of terrorist acts, followed by assailment, explosions, and hijackings. The data not only identify the order of occurrence but also emphasize the relative frequency of each type, contributing to a comprehensive understanding of the terrorism landscape. It therefore provides much more insights than the simple statistics revealed by the historical data. It is important to acknowledge that different regions of the world may see distinct patterns of terrorist acts. The above findings only reflect a global viewpoint and cannot be generalized to all regions. For instance, hostage taking tends to be more prevalent in Southeast Asia, whereas explosions are more frequently observed in the Middle East and North Africa. Based on the results, it can be inferred that BN outperforms basic statistical analysis by adeptly capturing intricate relationships and dependencies among variables, yielding a comprehensive system understanding. Furthermore, BN is exceptional at handling uncertainty, incorporating prior knowledge, and continuously updating beliefs. This capability improves decision‐making by providing nuanced insights that are often missed by more straightforward statistical methods.

**TABLE 3:** Comparative analysis of historical and Tree‐Augmented Naïve Bayes (TAN) results.

Attack type	Historical data (%)	TAN results (%)	Accuracy rate (%)
Assailment	24.5	24.7	99.2
Explosion	23.9	24.0	99.6
Hijacking	22.1	22.0	99.5
Hostage taking	29.5	29.3	99.3

#### 4.2.2 Data splitting and metrics analysis

In order to enhance the thorough evaluation of the model and accurately assess its predictive capabilities, a deliberate decision was made to randomly set aside 10% of the original database. This reserved portion, strategically chosen, was then utilized to rigorously test the model's performance. This testing phase took place after the model undergoing training, allowing for a comprehensive examination of how well the model generalized to new data and accurately predicted outcomes beyond its training set. As outlined in Table 4, the confusion matrix shows that the overall accuracy of the model stands impressively at 94.1%. Notably, when it comes to predicting assailment, the accuracy is slightly lower at 75%. However, for the prediction of other types of attacks, the model achieves a perfect accuracy rate of 100%. This high accuracy across various attack types highlights the robustness and reliability of the developed model in accurately classifying and predicting different forms of attacks.

**TABLE 4:** Confusion matrix of predicted results.

		Actual					Accuracy rate (%)
		Assailment	Explosion	Hijacking	Hostage taking	Total	
Predicted	Assailment	3	1	0	0	4	75.0
	Explosion	0	4	0	0	4	100
	Hijacking	0	0	4	0	4	100
	Hostage taking	0	0	0	5	5	100
	Total	3	5	4	5	17	94.1

In accordance with the details outlined in Section 3.4.3, Table 5 presents various performance metrics for each attack type based on the analysis of the confusion matrix. The “precision” of the model is exceptionally high, registering 100% for all attack types except for explosions, where it stands at 80%. Regarding “recall,” assailment exhibits a value of 75%, whereas other attack types achieve a perfect 100%. *F*‐measure, a composite metric of “precision” and “recall,” exceeds 85% across all categories. Notably, higher specificity contributes to enhanced model robustness. Specifically, assailment, hijacking, and hostage taking boast a specificity of 100%, whereas explosions, though slightly lower at 92%, still indicate substantial robustness. The FPR, inversely related to specificity, aligns with its patterns. An examination of these performance metrics underscores the commendable reliability and robustness of the developed model.

**TABLE 5:** Performance results for each Security Risk–Influencing Factor (SRIF).

	Precision (%)	Recall (%)	*F*‐measure (%)	Specificity (%)	FPR (%)
Assailment	100	75	86	100	0
Explosion	80	100	89	92	8
Hijacking	100	100	100	100	0
Hostage taking	100	100	100	100	0

An additional metric employed to evaluate the model's reliability is the “kappa coefficient,” also referred to as Cohen's kappa (Cohen, 1960). This metric measures the agreement between two raters or observers categorizing items. In our context, it quantifies the agreement between the predicted and actual results. Using the values derived from the confusion matrix (with the expected proportion of agreement equating to 0.25 and the observed proportion of agreement, representing overall accuracy, equaling 0.94), the kappa coefficient is determined to be 0.92. As a well‐established criterion suggests, a model is deemed nearly perfect when the kappa coefficient exceeds 0.8 (Landis & Koch, 1977). In our case, the model demonstrates robust consistency given its kappa coefficient of 0.92, indicating a high level of agreement beyond what would be expected by chance.

#### 4.2.3 Mutual information

The mutual information among the attack types, acting as the target node, and other SRIFs has been computed and is illustrated in Table 6, along with the associated entropy reduction percentage and variance of beliefs. Section 3.4.4 provides a comprehensive understanding of mutual information, with the corresponding variation signifying the difference among consecutive mutual information values. A higher mutual information value implies a more substantial impact of the respective SRIF on the attack type. Notably, the analysis reveals that the weapon type exerts the most significant influence on the attack types, accounting for nearly 11%. Following closely are the year, ship type, region, country, and perpetrator, contributing entropy reduction percentages of 7.21%, 5.56%, 5.19%, 4.45%, and 3.61%, respectively. This insight underscores the varying degrees of impact these factors have on determining the nature of attacks, with the weapon type playing the most pivotal role.

**TABLE 6:** Mutual information between attack type and Security Risk–Influencing Factors (SRIFs).

Node	Mutual information	Percentage (%)	Variance of belief
Attack type	1.99184	100	0.5589422
Weapon	0.21278	10.7	0.0215904
Year	0.14370	7.21	0.0166978
Ship type	0.11074	5.56	0.0141138
Region	0.10341	5.19	0.0112710
Country	0.08856	4.45	0.0109211
Perpetrator	0.07184	3.61	0.0096693
Property damage	0.05053	2.54	0.0055830
Successful attack	0.03175	1.59	0.0027943
Casualties	0.01969	0.988	0.0017811
Ransom	0.01835	0.921	0.0015514
Flag state	0.00955	0.479	0.0010733

#### 4.2.4 The joint probability and TRI calculation

Additional sensitivity methods are employed to validate the developed model and pinpoint the most influential SRIFs in determining the likelihood of various attack types against maritime ships. The joint probability of the SRIFs and the target node is computed, as outlined in Section 3.4.4. By incrementally setting the probability of each state of each node to 100%, the corresponding values for the states of the target nodes are obtained. Table 7 illustrates the results of the joint probability for the top six SRIFs identified from mutual information analysis. It is evident that, for different SRIF states, the values for target node states undergo changes compared to the original values. Examining the results in Table 7, the highest and lowest values for each attack type corresponding to the SRIF states are highlighted in bold for use in TRI calculation. Valuable insights can be derived from these results. For instance, the probability of an explosion is highest when explosive‐laden boats are used in terrorist attacks. For fishing boats, the likelihood of hostage taking is highest, particularly in the Southeast Asia region and in the country of Malaysia.

**TABLE 7:** The joint probability.

	Assailment	Explosion	Hijacking	Hostage taking
**Weapon**				
Bomb	21.6	47.9	13.0	17.5
Combinatory	**34.1**	25.7	17.4	22.8
Explosive‐laden boats	**14.0**	**60.5**	**12.4**	**13.0**
Firearms	29.4	**3.44**	25.6	**41.5**
Projectile	17.9	49.5	15.9	16.7
Unknown	17.9	23.4	**32.9**	25.8
**Ship type**				
Cargo ship	**16.6**	26.8	**40.9**	**15.7**
Civilian vessel	**41.8**	19.2	16.7	22.2
Commercial ship	20.8	24.0	36.2	19.1
Fishing boat	21.5	16.6	11.3	**50.6**
Other	36.3	32.0	**10.4**	21.4
Tanker	22.9	**34.5**	20.9	21.6
Tugboat	21.2	**16.2**	24.2	38.4
**Perpetrator**				
AA	21.5	20.9	26.4	31.2
ALQ	21.7	34.7	20.0	23.5
ALS	21.5	20.9	26.4	31.2
AMC	26.6	19.6	24.9	28.9
ASG	14.5	**11.9**	25.1	**48.5**
BIFM	**35.8**	21.0	19.9	23.4
FARC	33.0	19.6	18.6	28.9
HE	**14.3**	**53.0**	**13.3**	19.4
LTTE	25.1	34.2	18.7	22.0
ME	28.4	20.9	19.9	30.8
MEND	23.3	18.4	17.4	40.9
MMCM	26.3	19.4	18.4	35.9
NDV	28.4	20.9	19.9	30.8
NPA	23.3	30.0	21.5	25.2
Other	22.3	24.8	**34.4**	**18.4**
Unknown	34.9	19.3	20.1	25.7
**Country**				
Cameroon	26.6	19.6	24.9	28.9
Colombia	27.3	16.2	20.5	36.0
Congo	26.3	19.4	18.4	35.9
India	26.8	19.8	31.3	22.0
Iraq	23.1	22.5	21.3	33.1
Libya	18.8	18.3	28.9	34.1
Malaysia	22.9	**11.4**	**10.8**	**54.9**
Mozambique	20.0	19.5	24.7	35.8
Myanmar	21.5	20.9	26.4	31.2
Nigeria	**34.2**	12.3	20.9	32.6
Other	31.3	34.3	23.0	**11.5**
Philippines	**18.4**	20.3	29.5	31.8
Saudi Arabia	26.6	33.0	18.6	21.8
Somalia	18.9	18.4	**35.0**	27.6
Sri Lanka	25.1	34.2	18.7	22.0
Yemen	20.7	**52.2**	11.0	16.1
**Region**				
Middle East and North Africa	**21.0**	**48.8**	**12.6**	17.6
South America	27.9	18.4	21.0	32.7
South Asia	27.0	30.0	27.2	**15.9**
Southeast Asia	21.2	17.3	21.3	**40.3**
Sub‐Saharan Africa	**29.0**	**9.23**	**28.4**	33.3
**Year**				
2000	18.0	44.6	8.64	28.7
2001	30.2	9.96	38.5	21.3
2002	20.0	39.6	19.2	21.2
2003	20.0	39.6	19.2	21.2
2004	16.7	49.6	16.0	17.7
2005	39.9	19.8	19.1	21.2
2006	25.0	12.4	36.0	26.6
2007	33.3	22.0	21.2	23.5
2008	37.6	12.4	30.0	20.0
2009	36.1	17.9	17.3	28.7
2010	24.9	24.7	23.9	26.5
2011	32.9	16.3	15.8	35.0
2012	**39.9**	19.8	19.1	21.2
2013	24.7	32.6	7.88	34.9
2014	25.0	12.4	36.0	26.6
2015	33.1	32.8	10.6	23.4
2016	16.4	13.0	22.0	**48.7**
2017	13.3	**6.60**	**44.7**	35.4
2018	23.1	**53.3**	**7.36**	**16.3**
2019	28.9	11.5	16.6	43.0
2020	**12.5**	49.6	18.0	19.9
2021	22.3	22.1	32.0	23.6

**FIGURE 8:**
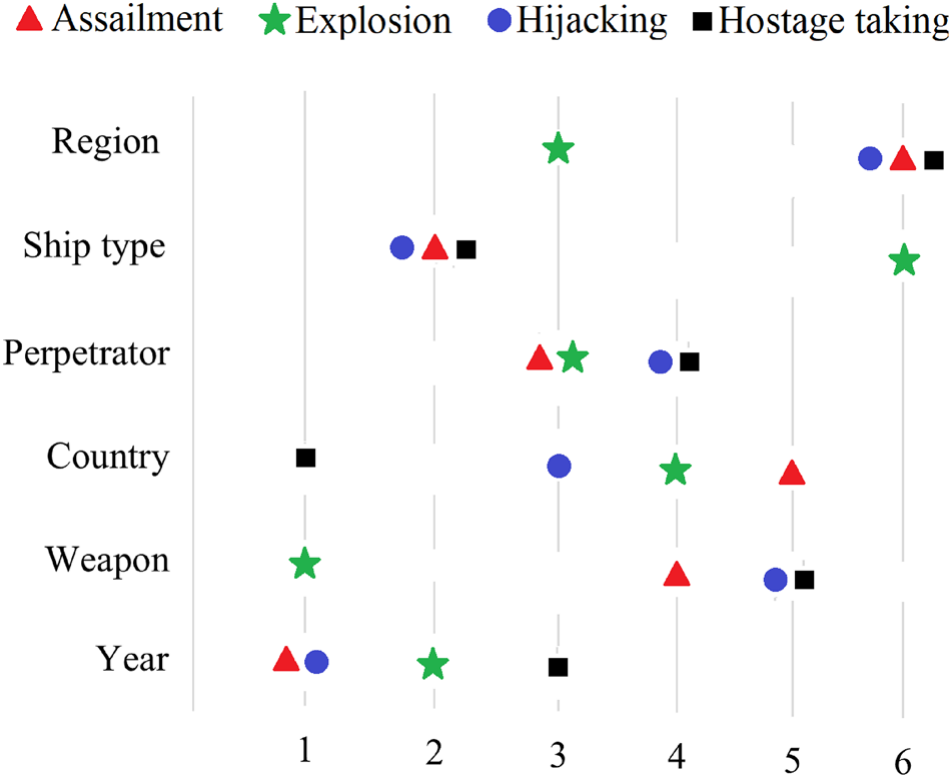
The ranking of Security Risk–Influencing Factors (SRIFs) for different types of attacks based on True Risk Influence (TRI) value.

In the context of TRI calculation, which is based on the insights from Section 3.4.4 and the outcomes of the joint probability analysis, the specific TRI values corresponding to all SRIFs have been computed and are presented in detail in Table 8. To shed light on the calculation process, this article focuses on TRI for the region, particularly in the case of explosions. Referring to the information in Table 7, it is observed that the Middle East and North Africa region contributed the most to the explosion, with a probability of 48.8%, whereas Sub‐Saharan Africa has the lowest contribution at 9.23%. When comparing these values with the original probability estimate for explosion (24%), the subsequent calculations for HRI and LRI are determined to be 24.8% and 14.77%, respectively. The TRI value, derived by averaging these calculated values, is computed to be 19.79%. The influence of various SRIFs on maritime terrorism is clearly dependent on the specific type of attack. Analyzing the average TRI across all attack categories reveals that the year emerges as the most significant factor, followed by weapon type, country, perpetrator, ship type, and region. Figure 8 illustrates the ordering of TRI values for SRIFs. This comprehensive approach to TRI not only highlights the varying contributions of different SRIFs but also provides a more detailed assessment of their impact on specific attack types, enhancing the interpretability of the model's findings.

**TABLE 8:** True Risk Influence (TRI) of Security Risk–Influencing Factor (SRIF) for different attack types.

	Assailment	Explosion	Hijacking	Hostage taking	Average
Year	13.70	23.35	18.67	16.20	17.98
Weapon	10.05	28.53	10.25	14.25	15.77
Country	7.90	20.40	12.10	21.70	15.53
Perpetrator	10.75	20.55	10.55	15.05	14.23
Ship type	12.60	9.15	15.25	17.45	13.61
Region	4.00	19.79	7.90	12.20	10.97

#### 4.2.5 Model verification

To validate the model further and adhere to the two principles outlined in Section 3.4.4, minor adjustments of 2% in the positive direction are applied to the prior probabilities of the most important identified variables. This adjustment is made in accordance with their importance levels determined through mutual information ranking. Subsequently, changes in the probability of the target node are observed. In Table 9, the initial probability of attack types is presented in the second column, whereas the subsequent columns depict their cumulative probabilities following an increase in the prior probabilities of other nodes. Notably, elevating the prior probabilities of SRIF nodes leads to a corresponding rise in the posterior probabilities of the target node. These outcomes align entirely with the specified principles, underscoring the robust validity of the TAN‐based BN model designed for analyzing maritime terrorism.

**TABLE 9:** The output of minor changes in Security Risk–Influencing FSactors (SRIFs).

Perpetrator	–	+2%	+2%	+2%	+2%	+2%	+2%
Country	–	–	+2%	+2%	+2%	+2%	+2%
Region	–	–	–	+2%	+2%	+2%	+2%
Ship type	–	–	–	–	+2%	+2%	+2%
Year	–	–	–	–	–	+2%	+2%
Weapon	–	–	–	–	–	–	+2%
**Assailment**	24.7	25.1	25.4	25.6	26.1	26.6	27.0
**Explosion**	24.0	24.8	25.7	26.5	26.9	27.9	29.1
**Hijacking**	22.0	22.4	22.9	23.2	23.9	24.7	25.1
**Hostage taking**	29.3	29.9	30.8	31.3	32.0	32.7	33.3

### 4.3 Real‐case scenario analysis

To reinforce the model's accuracy, two terrorist attack scenarios, which occurred recently and were not initially included in the original database, have been selected for testing. This deliberate selection of new, unseen data enhances the model's credibility by assessing its predictive capabilities on previously unencountered scenarios. The procedure operates by initially identifying specific SRIFs based on detailed information from reported incidents. These identified SRIFs are assigned a state with a probability of 100%. Subsequently, the probabilities of states for the target node are updated to reflect the predictive attack type.

**FIGURE 9:**
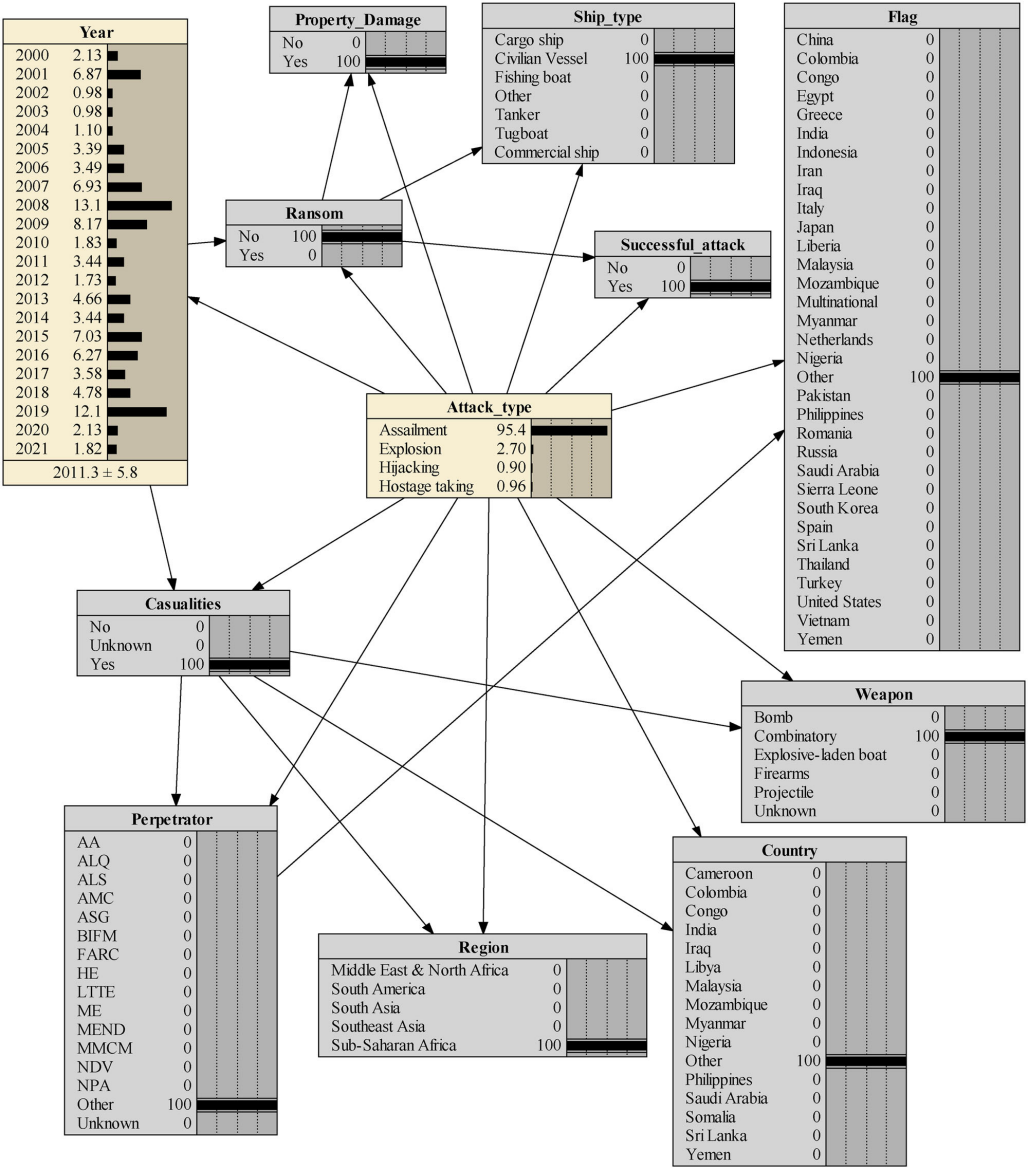
The first real‐case scenario analysis.

On September 8, 2023, a terrorist attack unfolded in Mali, located in the Sub‐Saharan Africa region, targeting a civilian marine vessel. The perpetrators, identified as the al‐Qaeda‐linked group, executed the attack using a combination of rockets and firearms. The incident took place on the River Niger as the boat traveled from Gao to Mopti. At least three rockets were launched at the vessel, specifically targeting its engines, resulting in the immobilization of the boat on the river. The military promptly initiated evacuation procedures for the passengers amidst an exchange of gunfire with the assailants. Unfortunately, the attack led to the deaths of 49 civilians and 15 soldiers. The terrorist attack was simulated using the constructed BN model, as depicted in Figure 9. The outcome indicates a remarkably precise prediction, with the incident probability accurately estimated at 95.4%.

**FIGURE 10:**
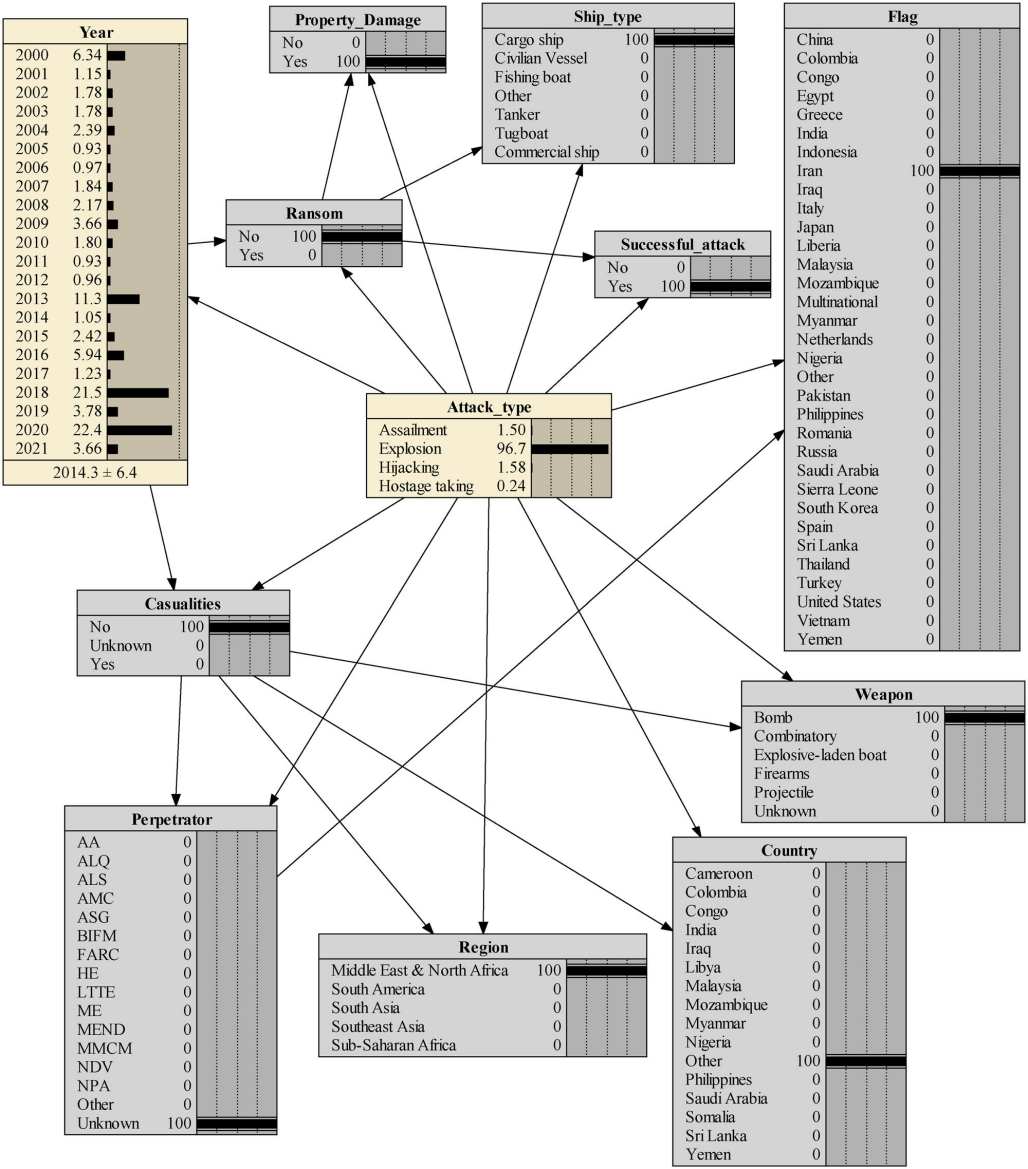
The second real‐case scenario analysis.

In another case, on March 10, 2021, an Iranian cargo ship fell victim to what has been described as a “terrorist” attack in the Mediterranean Sea. The vessel, owned by an Iranian state‐run company, was on its route from Iran to Europe when it was targeted by an explosive device, identified as a naval mine. The blast caused damage to the ship's hull, resulting in a small fire that was swiftly extinguished. Despite the intentional attack, there were no reported casualties. The initial findings suggest that the cargo ship was intentionally targeted by an unknown source, marking a deliberate act of violence against maritime transportation interests in the North African region. By assimilating the data from the report into the BN model, the model yielded a 96.7% probability of the occurrence of an explosion‐type attack, as demonstrated in Figure 10. This outcome underscores the model's precision in making highly accurate predictions.

### 4.4 Analytical discussion and implications

#### 4.4.1 Attack types

**FIGURE 11:**
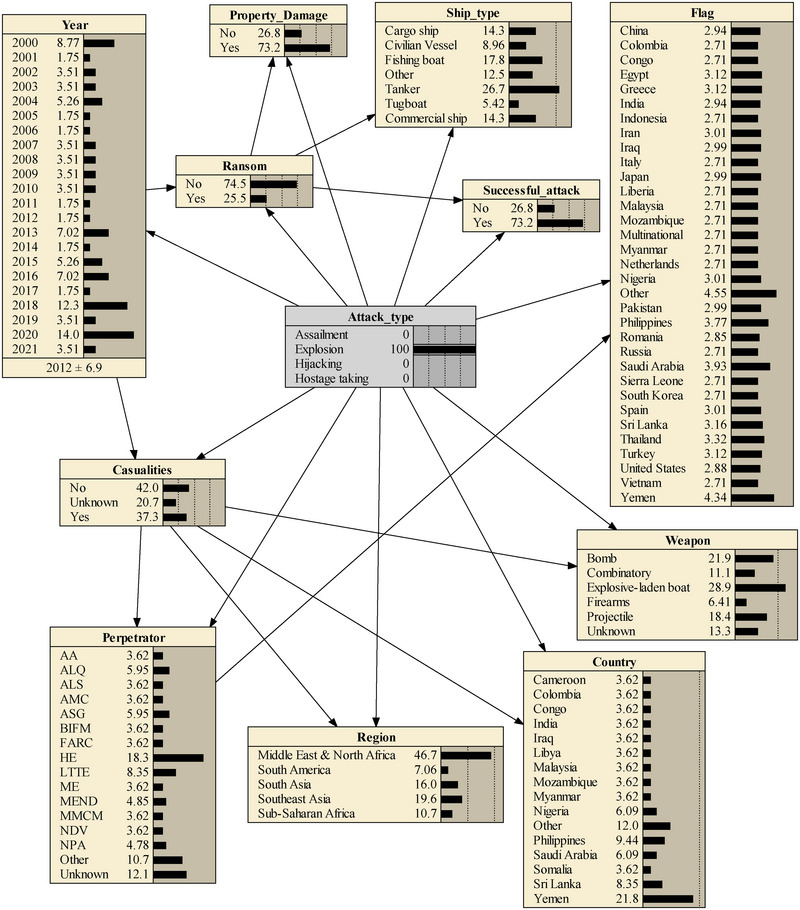
Explosion scenario.

The findings of the study led the authors to determine that each maritime transport region possesses unique characteristics related to maritime terrorism, categorizing them into types, such as assailment, explosion, hijacking, and hostage taking. Utilizing the constructed model and employing scenario analysis to investigate the influence of particular conditions on the target node allows for the extraction of valuable information. As an illustration, setting the explosion state in the target node to 100% can unveil the most probable scenario for an explosion incident. As depicted in Figure 11, several nodes show increased probabilities, indicating their contribution to this outcome. Regarding the type of weapon, explosive‐laden boats overwhelmingly precede other types, such as various bombs and projectiles, in terms of frequency of use. In reaction to the type of vessels, tankers are the most frequently targeted. Concerning high‐risk regions and countries, the Middle East and North Africa region, particularly Yemen as a country, emerge as the most perilous locations for the occurrence of explosion scenarios. This serves as a reminder of the tragic attack on the USS Cole, a U.S. Navy ship, in this region in 2000. The heightened risk can be attributed to the presence of HE (Houthi extremists) terrorist group with specific expertise in bombing tactics.

Similar data can be derived for various scenarios. In the context of hostage‐taking incidents, firearms are the predominant weapons used. Southeast Asia and Sub‐Saharan Africa emerge as the two high‐risk regions, with Malaysia, the Philippines, and Nigeria representing them, respectively. Fishing boats are commonly the preferred target for hostage‐taking scenarios. Regarding the perpetrating groups, Abu Sayyaf Group (ASG) is predominantly responsible for these incidents in Southeast Asia, showcasing meticulous expertise in both hostage taking and hijacking of vessels. Based on historical incidents, vessels flagged with Malaysia, Indonesia, and the Philippines are among the most frequently targeted by these groups, a trend not surprising given the geographical location of these ships in the mentioned region.

#### 4.4.2 Top SRIFs

According to the findings presented in Table 8, the top six SRIFs with the highest average TRI, listed in order, are “year,” “weapon type,” “country,” “perpetrator,” “ship type,” and “region.” The designation of “year” as the most influential risk factor suggests that the temporal dimension, specifically the particular year of an event, significantly influences the probability of maritime terrorist incidents. This designation can be viewed from multiple perspectives. Temporally, distinct periods of heightened and reduced terrorist activities have been observed over the past two decades. For instance, in 2016, more than 18% of all incidents took place, marking the year with the highest occurrence. Conversely, in 2002, 2003, 2005, and 2012, only one incident was recorded. Thus, certain years demonstrate discernible patterns or trends in the frequency of maritime terrorist incidents. From the standpoint of policy changes, it can be contended that post‐2016, the reinforcement of the military's defense strategy to prevent highly violent crimes in Philippine waters has contributed to a decline in the number of ASG members and the weakening of its units. This has resulted in a zero hostage‐taking rate in 2022. Another noteworthy aspect in this context is the progression of technology, encompassing both defensive and offensive capabilities, which has the potential to shape the methods and efficacy of terrorist attacks. Tracking the evolution of technology over time becomes crucial for anticipating potential threats. For instance, there is the possibility of retrofitting aquatic drones to operate as remotely controlled or autonomous waterborne improvised explosive devices, offering a discreet means to launch attacks against ships. Conversely, the development of advanced monitoring and detection security systems can play a pivotal role in mitigating the adverse impacts of such weaponry.

The type of weapon stands out as the second crucial SRIF with a substantial impact on the target node. It is evident that firearms play a pivotal role in most attack types, with a contribution of 60%, particularly in instances of hijacking and hostage taking. Regarding explosions, explosive‐laden boats and, in the case of assailment, combinatory weapons and projectiles emerge as the most influential states. This insight underscores the importance of early detection of weapon types, achievable either through intelligent services and coast guards or security monitoring devices on ships that can detect suspicious approaching boats. Such early detection measures can significantly contribute to neutralizing or, at the very least, mitigating the impact of these incidents.

The inclusion of “country” and “region” as the third and sixth top SRIFs underscores the pivotal role of these geographical aspects play in shaping the likelihood of diverse types of terrorist attacks. The recognition of a country as a risk factor acknowledges that the geopolitical landscape of a specific nation can exert a substantial influence on the occurrence and nature of maritime terrorist incidents. Notably, Yemen is recognized as a notorious country for explosion scenarios, the Philippines for hostage taking, and Nigeria for armed assault and hijacking. Various factors contribute to the varying risk profiles across countries, including political stability, governance effectiveness, and counterterrorism measures. These elements fluctuate widely, impacting the overall susceptibility of a country to maritime terrorism. The distinct geopolitical characteristics of each nation highlight the need for tailored security strategies that account for these specific contextual factors. Similarly, the region in which maritime activities unfold emerges as a critical factor. A nuanced understanding of regional dynamics is crucial for customizing security measures and response strategies to effectively address the specific challenges posed by maritime terrorism.

The identification of perpetrator groups as the fourth significant SRIF underscores their substantial impacts on various types of terrorist attacks. Distinct motivations, tactics, and capabilities among different perpetrators influence target selection, methods of attack, and overall implications for maritime security. Notorious terrorist groups, including ASG in the Philippines, HE in Yemen, Liberation Tigers of Tamil Eelam (LTTE) in Sri Lanka, and Movement for the Emancipation of the Niger Delta (MEND), are active in the context of maritime terrorism. Understanding the unique characteristics and behaviors of these groups is crucial for the development of targeted counterterrorism strategies.

The fifth SRIF is linked to the type of ships involved. Distinct ship categories face varying vulnerabilities, influencing the potential nature of attacks. Tankers, encompassing oil, gas, LNG, and chemical tankers, are particularly susceptible to explosive incidents, garnering widespread media attention. Cargo ships become targets for hijacking due to their valuable cargoes, serving as potential funding sources for terrorists. Fishing boats, characterized by easy accessibility, are prone to hostage‐taking scenarios. Civilian vessels, vulnerable to armed assaults, are targeted for heinous attacks with firearms. Addressing these diverse threats requires the implementation of tailored security measures, rules, and regulations across different maritime sectors.

#### 4.4.3 Implications

The generated implications highlight the need for a multifaceted approach involving various stakeholders to enhance maritime security and counter the evolving challenges posed by maritime terrorism, including the following:

(1) Governments and security agencies should recognize the significance of the temporal dimension (the specific year) in influencing the probability of maritime terrorist incidents. They should allocate resources and manpower, accordingly, focusing on years with discernible patterns of heightened activity.
(2) Policy changes and strategies should be adaptable based on temporal trends. For example, if certain years show increased activity, governments may need to reinforce defense strategies or law enforcement efforts during those periods.
(3) Continuous monitoring of technological advancements is vital for anticipating potential threats. Governments should invest in intelligence and research capabilities to stay ahead of evolving technologies that could be used in maritime terrorist attacks.
(4) The type of weapon used in attacks plays a significant role. Shipping companies should invest in advanced security measures, such as intelligent services, coast guards, and monitoring devices, to detect and respond to different weapon types, especially explosive‐laden boats, and firearms.
(5) Companies operating in different regions should conduct thorough risk assessments. In fact, understanding the geopolitical landscape of specific countries can help in tailoring security measures and response strategies to mitigate potential threats.
(6) Early detection of suspicious approaching boats is critical for the maritime industry. Companies should, therefore, invest in advanced monitoring and detection security systems to neutralize or mitigate the impact of incidents.
(7) Understanding the motivations, tactics, and capabilities of different perpetrator groups is essential. Counterterrorism agencies should focus on intelligence gathering and profiling of terrorist groups active in maritime terrorism.
(8) Targeted counterterrorism strategies that account for the unique characteristics and behaviors of specific terrorist groups should be developed. This includes tracking their activities, funding sources, and recruitment methods.
(9) Promote international collaboration and information sharing to combat maritime terrorism. Sharing data on temporal patterns, weapon types, and perpetrator groups can enhance global maritime security.
(10) Support capacity‐building initiatives in vulnerable regions. This includes assisting countries with political instability or governance challenges to improve their ability to counter maritime terrorism.
(11) Encourage regional cooperation in addressing maritime security challenges. Understanding regional dynamics and challenges is crucial for effective security measures.
(12) The defense industry should continue to innovate in both defensive and offensive capabilities to address evolving terrorist tactics. Developing advanced monitoring and detection systems can play a pivotal role in mitigating the impact of new weaponry.
(13) Invest in the research and development of countermeasures against potential threats involving aquatic drones or other emerging technologies. Developing technology to counter such threats can enhance maritime security.
(14) Civil society organizations should advocate for government policies that prioritize maritime security and counterterrorism efforts. They can also contribute to public education and awareness campaigns.

## 5 CONCLUSION

This article presents a novel contribution to maritime terrorism research, encompassing both qualitative and quantitative dimensions. The study draws upon incidents from the widely regarded GTD database, the primary and publicly accessible repository, covering the past two decades. From an initial pool of over 200 cases, meticulous refinement leaves approximately 160 incidents for in‐depth analysis, focusing specifically on terrorist attacks directed at maritime transportation. The investigation, fueled by both data analysis and an extensive literature review, identifies 12 SRIFs. Four distinct types of terrorist attacks—assailment, explosion, hijacking, and hostage taking—are chosen as target nodes for the study. To model and understand the complex interplay of these factors, a TAN‐based BN model is constructed using the curated dataset, facilitating a nuanced exploration of risk diagnosis and prediction in the realm of maritime terrorism. Delving into the model's analysis, it emerges that six SRIFs—namely, year, weapon type, country, perpetrator, ship type, and region—exert considerable influence on the selected target nodes. The robustness and validity of the model are thoroughly tested through an array of verification techniques, including comparative, metrics, sensitivity, and real‐case scenario analyses. Impressively, the results attest to the model's high level of reliability. Taking the investigation, a step further, the model is subjected to real‐world scrutiny by testing it against two recent terrorist attacks not included in the training dataset. Remarkably, the model predicts the attack types with over 90% probability accuracy in both cases, showcasing its applicability to real‐world scenarios and its potential as a valuable tool for risk assessment and prediction in maritime security. The study's findings offer valuable insights for individuals and government entities, enhancing understanding of maritime terrorism and potentially strengthening preventive measures and emergency management. Additionally, the research establishes a reliable foundation for collaborative counterterrorism initiatives across various countries and regions.

In considering future research directions, one could explore the comparison of terrorist attacks in the maritime sector with those in other modes of transportation, such as aviation, rail, and road systems. The objective would be to identify potential correlations and commonalities among them. Additionally, it is crucial to assess the alignment of existing maritime industry policies, such as the ISPS code, with the actual security needs and their effectiveness in countering terrorist activities.

## CONFLICT OF INTEREST STATEMENT

The authors declare that they have no known conflicts of interest or personal relationships that could have appeared to influence the work reported in this article.
